# Supplementary material for: Molecular Epidemiology of Mycobacterium tuberculosis Complex Strains in Urban and Slum Settings of Nairobi, Kenya
Source: Genes (Basel). 2022 Mar 8;13(3):475. doi: 10.3390/genes13030475 (PMC8953814; doi:10.3390/genes13030475)
Supplement: Supplementary file 1 [file genes-13-00475-s001.zip › genes-1608183 Supplementary Figures.pdf]

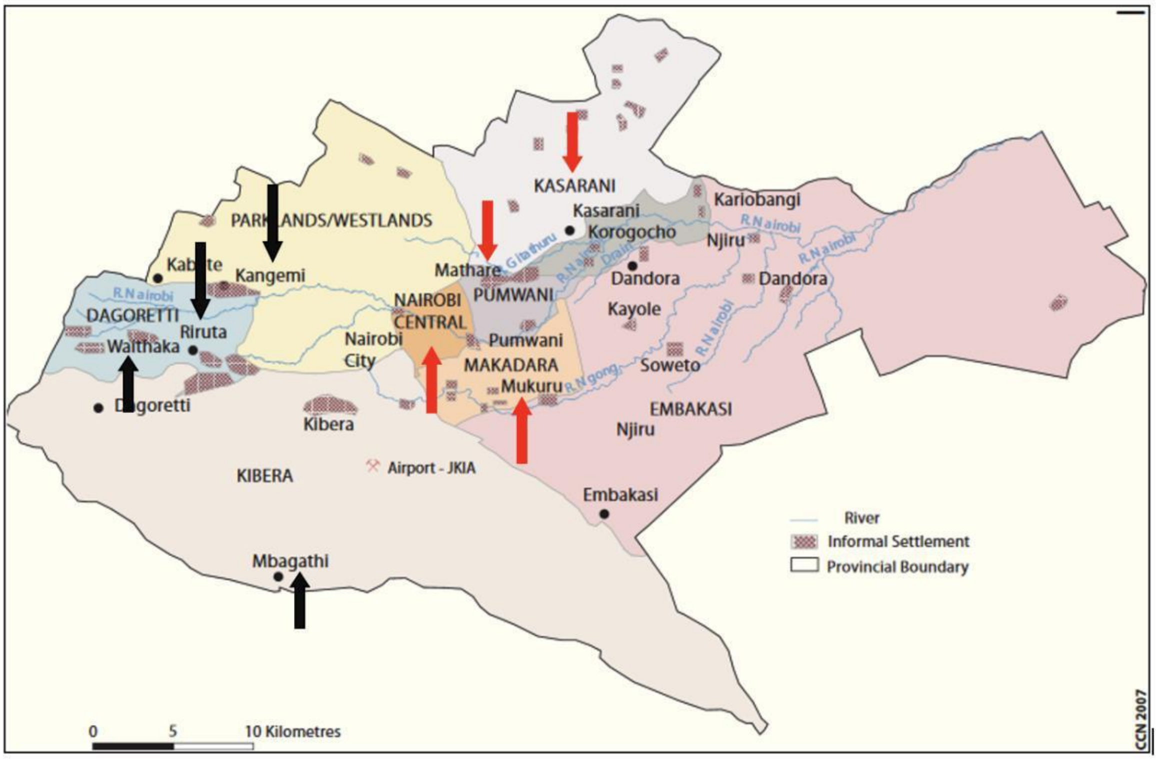

Figure S1. Sectional map of Nairobi, Kenya.

Black arrows indicate study sites in cohort A (2010) and red arrows indicate study sites in cohort B (2014–2015).

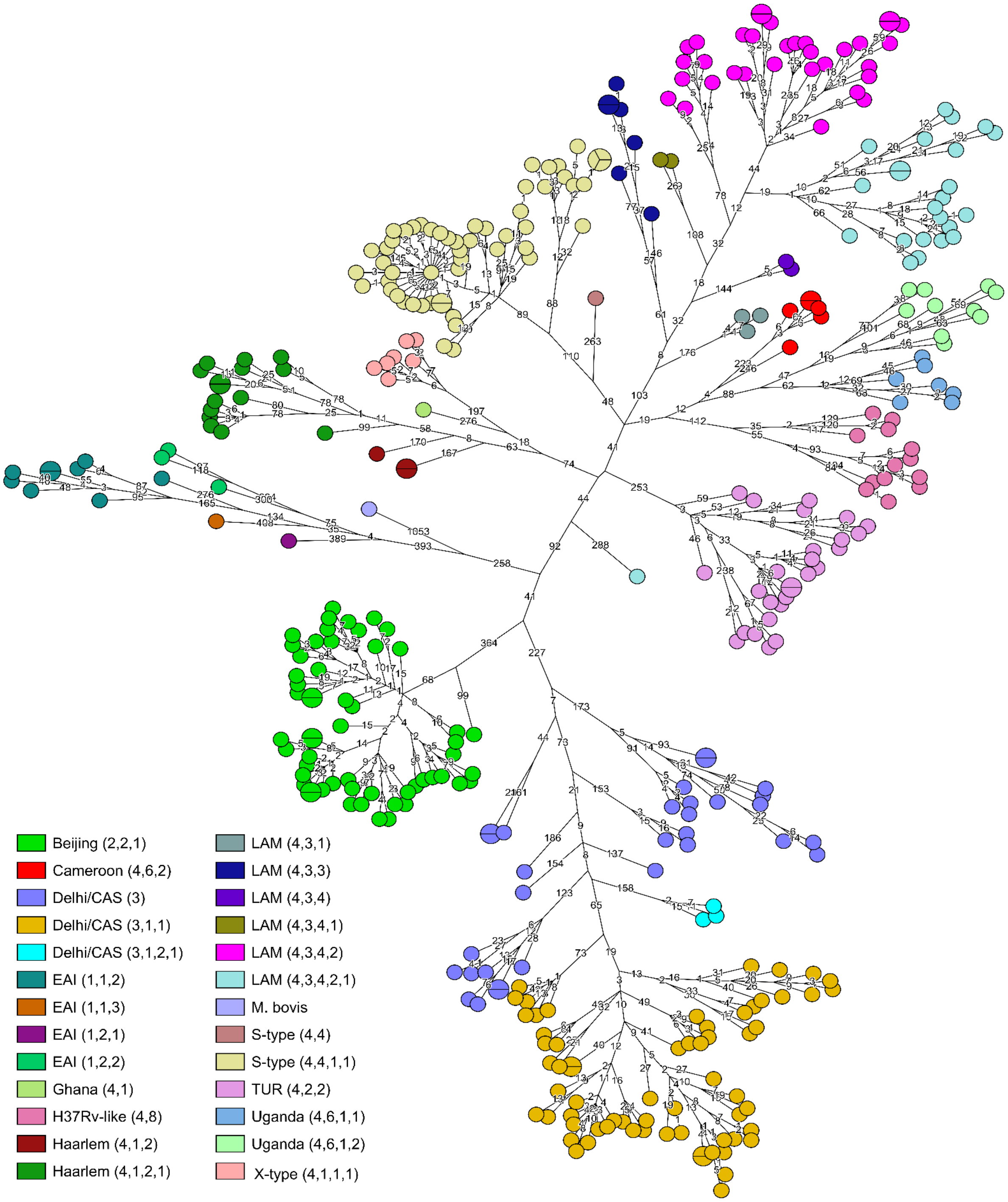

Figure S2. Phylogenetic tree showing MTBC lineage classification of all analyzed MTBC isolates in urban and slum settings of Nairobi, Kenya.

The phylogenetic tree based on maximum parsimony was calculated based on 18,167 concatenated SNPs showing the identified *M. bovis* and MTBC lineages (L1–4). The genetic distance (SNP differences) is indicated on tree branches. Different lineages and sublineages are indicated in different colors with an SNP bar code. MTBC, *Mycobacterium tuberculosis* complex; SNP, single-nucleotide polymorphism.

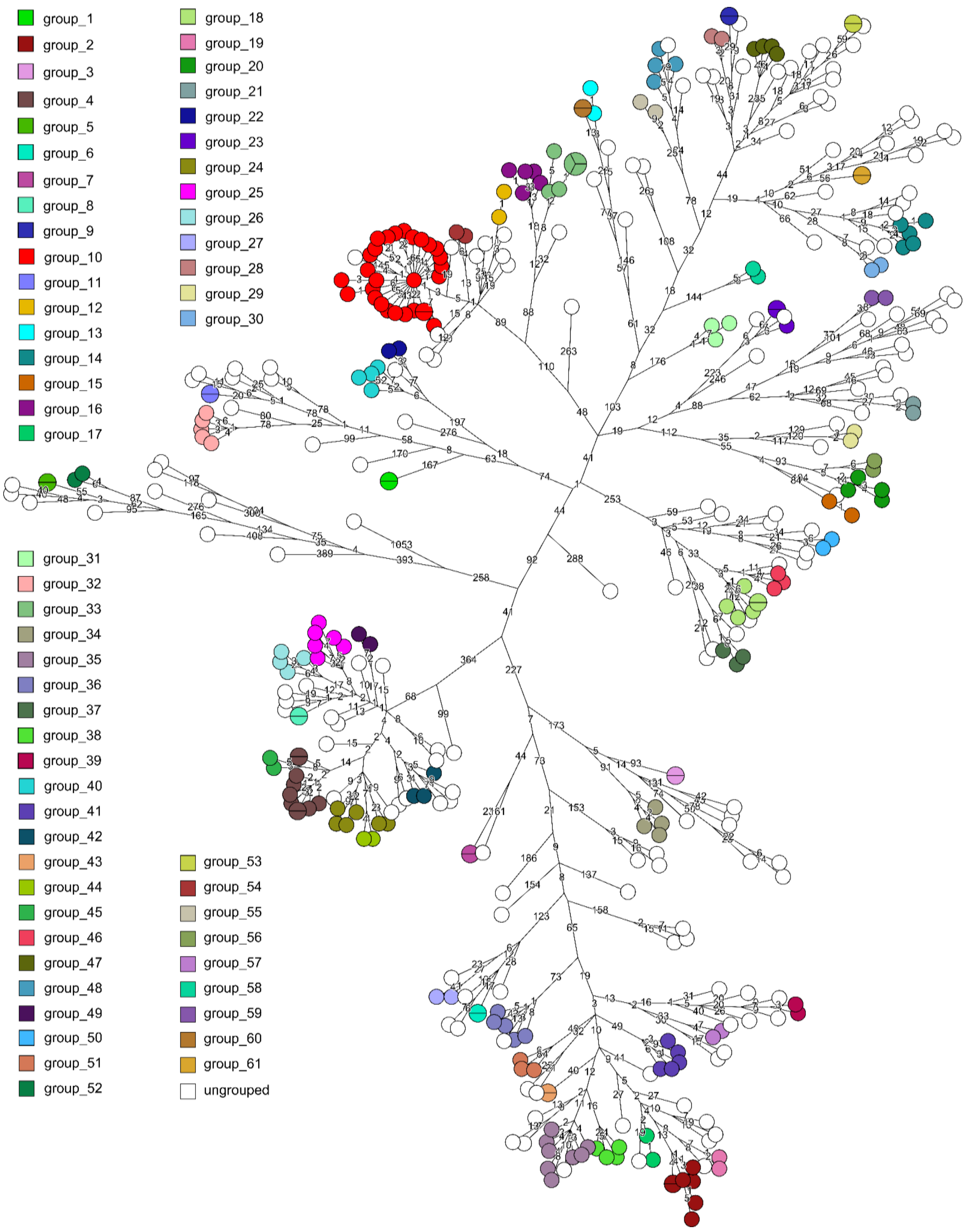

Figure S3. Phylogenetic tree showing <12 SNP clusters in urban and slum settings of Nairobi, Kenya.

The phylogenetic tree based on maximum parsimony was calculated based on 18,167 concatenated SNPs showing molecular clusters. The genetic distance (SNP differences) is indicated on tree branches. Different clusters are indicated in different colors. SNP, single-nucleotide polymorphism.

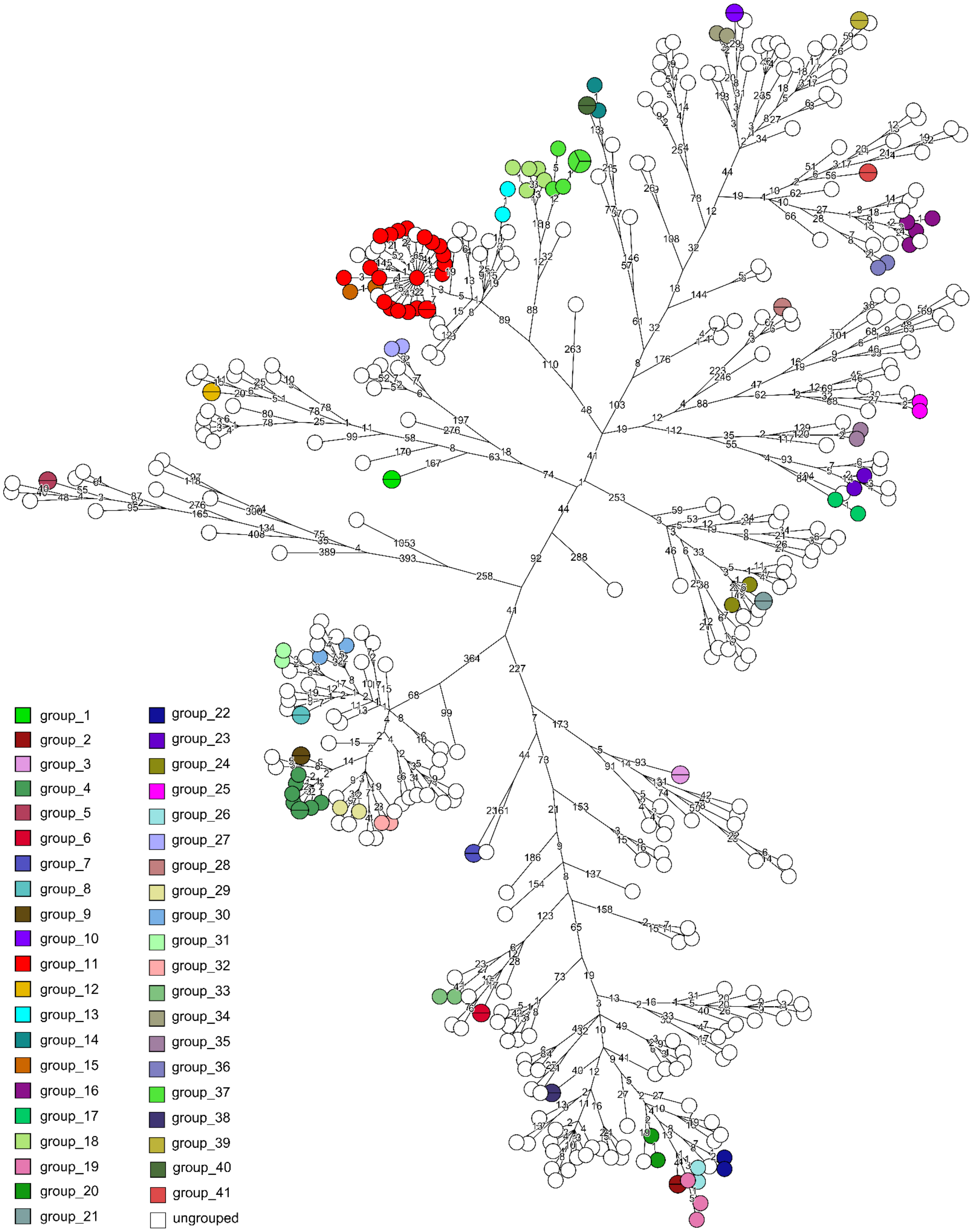

Figure S4. Phylogenetic tree showing <5 SNP clusters in urban and slum settings of Nairobi, Kenya.

The phylogenetic tree based on maximum parsimony was calculated based on 18,167 concatenated SNPs showing molecular clusters. The genetic distance (SNP differences) is indicated on tree branches. Different clusters are indicated in different colors. SNP, single-nucleotide polymorphism.

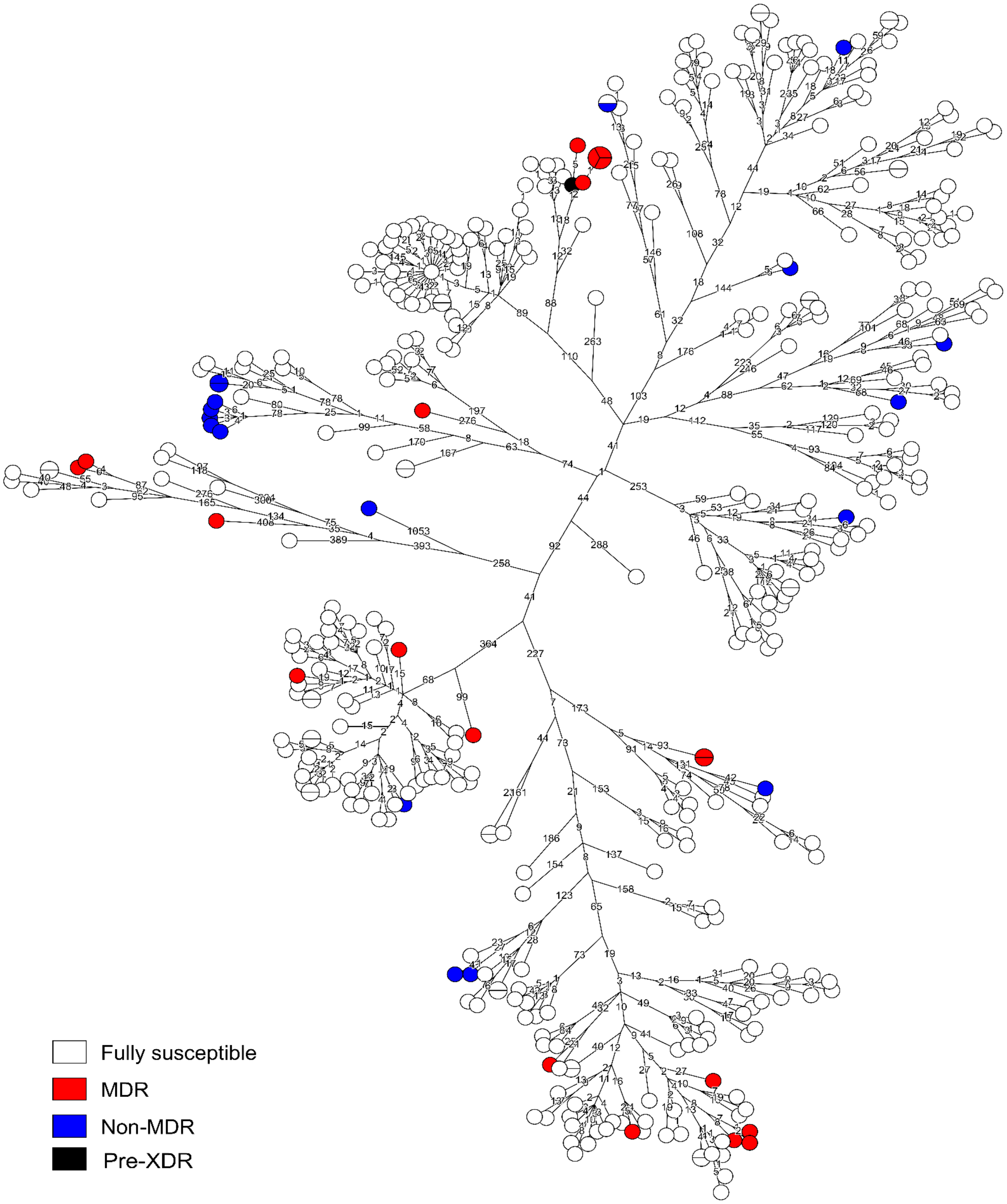

Figure S5. Phylogenetic tree showing genotypic drug resistance in urban and slum settings of Nairobi, Kenya.

The phylogenetic tree based on maximum parsimony was based on 18,167 concatenated SNPs showing strains that harbor drug resistance mutations (non-MDR, MDR, and pre-XDR isolates) and wild type (i.e., isolates with no drug resistance mutations). The genetic distance (SNP differences) is indicated on the branches. The largest cluster comprises six L4.4.1.1 (S-type) (i.e., five MDR and one pre-XDR) strains that shared the following resistance-conferring mutations: *katG* Ser315Thr, *rpoB* Ser450Leu, *pncA* Lys96Thr, *rpsL* Lsy43Arg, and *embB* Met306Ile. The pre-XDR strain of this cluster harbors an additional mutation (Asp94Gly) towards fluoroquinolones in the *gyrA* gene.
